# Changes in behavior are unable to disrupt a trophic cascade involving a specialist herbivore and its food plant

**DOI:** 10.1002/ece3.5118

**Published:** 2019-04-08

**Authors:** Madeleine G. Lohman, Thomas V. Riecke, Cheyenne R. Acevedo, Brian T. Person, Joel A. Schmutz, Brian D. Uher‐Koch, James S. Sedinger

**Affiliations:** ^1^ Department of Natural Resources and Environmental Science University of Nevada‐ Reno Reno Nevada; ^2^ Program in Ecology, Evolution, and Conservation Biology University of Nevada‐Reno Nevada; ^3^ Department of Wildlife Management North Slope Borough Barrow Alaska; ^4^ U.S. Geological Survey, Alaska Science Center Anchorage Alaska

**Keywords:** behavior, black brant, *Carex subspathacea*, growth, population dynamics, survival

## Abstract

Changes in ecological conditions can induce changes in behavior and demography of wild organisms, which in turn may influence population dynamics. Black brant (*Branta bernicla nigricans*) nesting in colonies on the Yukon–Kuskokwim Delta (YKD) in western Alaska have declined substantially (~50%) since the turn of the century. Black brant are herbivores that rely heavily on *Carex subspathacea* (Hoppner's sedge) during growth and development. The availability of *C. subspathacea* affects gosling growth rates, which subsequently affect pre‐ and postfledging survival, as well as size and breeding probability as an adult. We predicted that long‐term declines in *C. subspathacea* have affected gosling growth rates, despite the potential of behavior to buffer changes in food availability during brood rearing. We used Bayesian hierarchical mixed‐effects models to examine long‐term (1987–2015) shifts in brant behavior during brood rearing, forage availability, and gosling growth rates at the Tutakoke River colony. We showed that locomotion behaviors have increased (*β* = 0.05, 95% CRI: 0.032–0.068) while resting behaviors have decreased (*β* = −0.024, 95% CRI: −0.041 to −0.007), potentially in response to long‐term shifts in forage availability and brood density. Concurrently, gosling growth rates have decreased substantially (*β* = −0.100, 95% CRI: −0.191 to −0.016) despite shifts in behavior, mirroring long‐term declines in the abundance of *C. subspathacea* (*β* = −0.191, 95% CRI: −0.355 to −0.032). These results have important implications for individual fitness and population viability, where shifts in gosling behavior putatively fail to mitigate long‐term declines in forage availability.

## 1 INTRODUCTION

Individual fitness and population dynamics can be driven by resource availability (Oro, Cam, Pradel, & Mertinez‐Abrain, 2004) and environmental conditions during growth and development (Cooch, Rockwell, & Brault, 2001; Sedinger, Flint, & Lindberg, 1995; Sedinger et al., 2001,2016). When environmental conditions change, organisms may alter their behavior in an attempt to maintain fitness (Anholt & Werner, 1995; Charmantier et al., 2008). However, when extreme changes in resource availability occur, organisms may be unable to modify their behavior sufficiently to compensate for declining resources (Moller, Rubolini, & Lehikoinen, 2008; Walther et al., 2002). Such changing conditions can lead to reductions in individual fitness (Nussey, Postma, Gienapp, & Visser, 2005; Williams, Cooch, Jefferies, & Cooke, 1993) and population declines (Both, Bouwhuis, Lessells, & Visser, 2006; Moller et al., 2008). Shifts in resource availability and quality are especially important during the development of long‐lived species, as growth of juveniles can affect the probability of survival past the first year of life (Schmutz, 1993), adult body size (Cooch, Lank, Rockwell, & Cooke, 1991), and future fecundity (Lindstrom, 1999; Sedinger, Flint, et al., 1995; Sedinger, Herzog, & Ward, 2004). Such challenges are enhanced for migratory species, where energy reserves are often required to make long journeys shortly after the growth period (Jehl, 1997; Piersma, 1998; Schmutz, 1993), resulting in substantial mortality during the first migration (Menu, Gauthier, & Reed, 2005; Owen & Black, 1989). Consequently, the quantity and quality of food available during the growth period are critical to first‐year survival, future fitness, and population growth rates of long‐lived migratory species (Fondell, Flint, Sedinger, Nicolai, & Schamber, 2011; Francis, Richards, Cooke, & Rockwell, 1992; Owen & Black, 1989; Sedinger & Chelgren, 2007; Sedinger et al., 2016).

Populations of black brant (*Brant bernicla nigricans*; hereafter, brant) breeding in major colonies on the Yukon–Kuskokwim Delta, Alaska (hereafter, YKD) have declined by more than 50% since the turn of the 21st century (Wilson, 2016). Observed declines have been attributed to extensive reproductive failures due to nest predation by Arctic foxes (*Vulpes lagopus*; Sedinger et al., 2016), as well as long‐term declines in first‐year and adult survival, as well as female condition (Van Dellen, 2016; Leach et al., 2017).

These conservation issues concerning fox predation and colony declines may be linked to those of forage availability (Sedinger et al., 2016). Brant populations exhibit complex relationships with their primary food source during growth and development, *Carex subspathacea* (Hoppner's sedge). When grazing pressure is sufficiently increased, a result of high nest density and nest survival, *C. subspathacea* grazing lawns, which are higher in nitrogen concentration than surrounding graminoid communities, are maintained or expanded (Person et al., 2003; Ruess, Uliassi, Mulder, & Person, 1997). Additionally, during and after grazing, these lawns are fertilized by goose feces, increasing the deposition and subsequent recycling of nitrogen and other nutrients by the sedge (Bazely & Jefferies, 1985; Ruess et al., 1997). However, during extensive fox predation events on nests, grazing pressure is reduced, as most adults migrate to higher latitude molting areas following reproductive failure and breeding colony densities decline (Bollinger & Derksen, 1996). When reductions in grazing pressure occur, grazing lawn vegetation reverts to a taller and less nutritious growth form (Person et al., 2003, Uher‐Koch et al., unpublished data) which is insufficient to support gosling growth (Herzog & Sedinger, 2004) and is not preferred as a forage species by brant (Person et al., 2003).

Reduced nutrient availability leads to reduced gosling growth rates (Lindholm, Gauthier, & Desroches, 1994; Sedinger et al., 2001), which in turn affects pre‐ and postfledging survival, as well as body size and breeding propensity as an adult (Sedinger & Chelgren, 2007; Sedinger, Flint, et al., 1995). Declines in recruitment and reproductive effort of adults originating from cohorts with poor growth (Cooch et al., 2001; Sedinger & Chelgren, 2007) negatively influence population dynamics (Sedinger, Flint, et al., 1995; Sedinger & Nicolai, 2011; Sedinger et al., 2016). Given a short subarctic growing season and long‐distance migratory behavior, goslings must rapidly acquire and process nutrients to develop and fledge (Schmutz, 1993; Sedinger, Flint, et al., 1995), and accordingly, they spend about 70%–75% of their time foraging (Sedinger, Eichholz, & Flint, 1995). When there is less per capita forage available, as in years of high population density, foraging time has been observed to increase, enabling the acquisition of nutrients necessary for growth and survival (Sedinger, Eichholz, et al., 1995; Sedinger et al., 2016). Similarly, broods may also alter their foraging strategies and behaviors to gain the necessary nutrients for growth and migration when food abundance is reduced, as in years with reduced lawn extents resulting from reduced grazing pressure. Moreover, with increasing evidence of phenological mismatches in Arctic systems, timing of peak hatch dates for goslings may occur after the height of nutrient availability in grazing lawns, which may already be declining, decreasing recruitment further (Post & Forchhammer, 2000; Ross, Alisauskas, Douglas, & Kellett, 2017).

As food abundance in brood‐rearing areas on the YKD may have decreased as a consequence of potential declines in grazing pressure, we expected changes in demographic components of resident species in these habitats, as well as behavioral shifts. We hypothesized that the size of goslings near fledging would decline in response to reduced availability of *C. subspathacea* due to reduced grazing lawn extent from low population densities and that brood behavior would also vary in an attempt to buffer and respond to the effects of reduced food availability. Specifically, we predicted that primary changes in behavior would include increases in the proportion of time spent foraging by broods to respond to reduced grazing lawn extent resulting from lower brood densities. We also projected that adults would reduce time spent engaging in alert behaviors, which serve to reduce predation risk of their offspring (Lazarus & Inglis, 1978), at lower levels of food abundance as a result of increased time spent foraging (Sedinger, Eichholz, et al., 1995). Evidence for behavioral shifts could have important implications for understanding the ability of long‐lived specialist organisms to respond to habitat alteration or degradation.

## 2 MATERIALS AND METHODS

### 2.1 Study area

We conducted the study on the Tutakoke River brant colony (TRC) (61.25°N, 165.61°W) and associated brood‐rearing areas (Lindberg, Sedinger, Derksen, & Rockwell, 1998; Nicolai & Sedinger, 2012) on the YKD near the mouth of the Kashunuk River. Communities of *C. subspathacea* and *Puccinellia phryganodes* are only a few centimeters above mean high tide and patchily cover the landscape along the margins of coastal mudflats and around ponds and lakes (Jorgenson, 2000; Person, Babcock, & Ruess, 1998). A few centimeters higher in elevation, the vegetation quickly shifts to communities dominated by *C. ramenskii, Elymus arenarius, Potentilla edgedii,* and *Triglochin palustris* (Jorgenson, 2000). *C. subspathacea* appears to be a grazed morph of *C. ramenskii* as the two can be freely interconverted by natural grazing or experimental mowing (Person et al., 2003).

### 2.2 Gosling growth

Black brant breeding at the TRC have been studied intensively since 1984, and this study includes gosling growth data from 1987 to 2014. To estimate gosling growth and survival rates, goslings in nests of previously marked adults (see below) were tagged within 1 day of pipping with a uniquely marked fish fingerling tag (Alliston, 1975; Sedinger, Eichholz, et al., 1995). Because hatching requires about 24 hr, age and hatch dates are accurate to ±1 day (Sedinger, Flint, et al., 1995). Following hatch, adults and their goslings departed within 24 hr to brood‐rearing areas up to 25 km away (Nicolai & Sedinger, 2012). Goslings were then recaptured with parents at 22–45 days of age during the adult remigial molt by herding them into corral traps (Sedinger, Lindberg, Rexstad, Chelgren, & Ward, 1997). During this time, goslings and untagged adults were banded with uniquely engraved 2.5‐cm‐tall plastic bands and U.S. Geological Survey steel bands. Previously marked adults were recorded and measured. Sex of tagged goslings was determined by cloacal examination (Owen, 1980); gosling were then weighed and measured on an electronic balance (Fondell et al., 2011; Sedinger, Eichholz, et al., 1995).

### 2.3 Brood behavior observations

Brood behavior observations took place from 1987 to 2015. To study brood behavior, observers stayed in three to seven meter high towers fitted with observation blinds (Sedinger, Eichholz, et al., 1995). Towers were entered at around 22:00 hour, and observations began the next morning to allow brood behavior to return to normal patterns after the disruption of entering the blind (Sedinger, Eichholz, et al., 1995). Observers remained in the blinds for 1–3 days to minimize disturbance to broods and behaviors responding to human presence (Sedinger, Eichholz, et al., 1995). Broods associated with color‐banded adults were opportunistically selected for observation. Behaviors of males, females, and goslings were recorded every minute for an hour, the duration of a full observation bout. One hour represented the approximate time required for a brood to exhibit a full range of common behaviors (Sedinger, Eichholz, et al., 1995). Only observation bouts lasting over 30 min were considered in analyses, as some observations did not last the full hour if broods moved out of sight. We classified behaviors as foraging (head down and either searching for, or pecking at, food), alert (sitting and standing), walking (without foraging), aggression, and resting (Sedinger & Raveling, 1988). We distinguished adult males from females based on body size and plumage (Sedinger, Eichholz, et al., 1995); additionally, many observed individuals had been previously marked and sexed. It was not possible to assign sex to individual goslings, nor was it possible to follow individual goslings, so behavior during each focal sample was assigned to the behavior exhibited by the majority of goslings in the focal brood (Sedinger, Eichholz, et al., 1995).

Behavioral patterns may vary similarly between adults and juveniles, as broods act as a family unit, foraging, walking, and resting at similar times. In searching for acceptable quality forage, locomotion can be indicative of the abundance and extent of grazing lawns, with more travel required between forage patches when abundance is low (Norberg, 1977). Low abundance of resources may induce competition for high‐quality grazing lawns, where adults engage in aggression to defend foraging territories (Mulder, Williams, & Cooke, 1995; Sedinger, Eichholz, et al., 1995). When food is sufficiently abundant, rest may be indicative of a digestive bottleneck, where goslings can ingest food faster than it can be digested, and must periodically pause foraging (Kersten & Visser, 1996; Sedinger & Raveling, 1988). Alert behaviors by adults may be an attempt to mitigate predation risk while offspring are vulnerable (Lazarus & Inglis, 1978). The proportion of time spent exhibiting aggression and vigilance is influenced by sex; males spend more time exhibiting these behaviors as females must forage to regain mass after incubation periods (Sedinger, Eichholz, et al., 1995; Sedinger & Raveling, 1990).

### 2.4 Grazing lawn extent

Aerial videography and photography surveys have been conducted to quantify grazing lawn abundance at TRC since 1991 (Lake, Lindberg, Schmutz, Anthony, & Broerman, 2006; Person et al., 2003; B. D. Uher‐Koch, J. A. Schmutz, H. M. Wilson, R. M. Anthony, T. L. Day, B. T. Person, & J. S. Sedinger, unpublished data). We included data from Person et al. (2003), for the years 1991–1999, and B. D. Uher‐Koch, J. A. Schmutz, H. M. Wilson, R. M. Anthony, T. L. Day, B. T. Person, & J. S. Sedinger (unpublished data), 1993–2016, to increase sample size and spatial coverage. For 1991, 1993–1995, 1997–1999, 2001, 2004, and 2007–2016, we randomly selected ~250 photos or stills from videos each year for a total of 4,765 photographic or videographic samples. We subsequently overlaid 10 × 10 (1991–1999; data collected by Person et al. (2003) or 12 × 15 grids (1993–2016; data collected by B. D. Uher‐Koch, J. A. Schmutz, H. M. Wilson, R. M. Anthony, T. L. Day, B. T. Person, & J. S. Sedinger (unpublished data)) over each photographic sample and assigned each point within each sample to either *C. subspathacea* or other habitat, for a total of 723,119 habitat assignments. Photographs covered approximately 69 m^2^ of ground space, and each sampled photograph was spaced at least 200 m from other photographs (Lake et al., 2006; B. D. Uher‐Koch, J. A. Schmutz, H. M. Wilson, R. M. Anthony, T. L. Day, B. T. Person, & J. S. Sedinger, unpublished data). For further details on methods examining grazing lawn extent, please see Anthony, Anderson, Sedinger, and McDonald (1995), Person et al. (2003), Lake et al. (2006), and B. D. Uher‐Koch, J. A. Schmutz, H. M. Wilson, R. M. Anthony, T. L. Day, B. T. Person, & J. S. Sedinger (unpublished data).

### 2.5 Gosling growth rate analysis

To examine temporal variation in gosling growth rates, we constructed mixed‐effects linear models in JAGS (Plummer, 2003) using the R (R Core Team, 2018) package “jagsUI” (Kellner, 2018). Here and throughout, subscript (*t*) represents time (year) and (*i*) the individual. We modeled each individual's mass as a normal distribution with a predicted individual mean (*µ_i_*_,mass_) and population‐level variance (*σ* ^2^_mass_).

$${\text{mass}}_{i}∼\text{normal(}\mu_{i,\text{mass}},\sigma_{\text{mass}}^{2}).$$

We assumed a linear relationship between gosling age in days and mass (*β_t_*_,mass_), where we modeled year‐specific daily growth rates as a function of a random effect around a mean year‐specific growth rate (*µ_t_*_,growth_) with variance ($\sigma_{\text{growth}}^{2}$), and a trend across years on the mean of the random effect. The first alpha value (43.6) used was based on the mean mass of brant at hatching (Palmer, ). The second alpha value (*α_t_*_,growth_) describes the intercept for gosling mass at 30 days for each year. The second beta value (*β*_growth_) was the linear trend of gosling mass at 30 days among years.

$$\mu_{i,\text{mass}}=43.6+\beta_{t,\text{mass}}*{\text{age}}_{i}.$$

$$\beta_{t,\text{mass}}∼N\left(\mu_{t,\text{growth}},\sigma_{\text{growth}}^{2}\right).$$

$$\mu_{t,\text{growth}}=\alpha_{t,\text{growth}}+\beta_{\text{growth}}*\text{year}.$$

We acknowledge growth rates are not linear. However, gosling growth is approximately linear across the range of ages at which we captured goslings (Hupp et al., 2017; Lindholm et al., 1994; Sedinger, Eichholz, et al., 1995), so linear models were used in the interest of parsimony. Gosling growth rates are confounded with intra‐annual variation in hatch date, and attempting to fit nonlinear models would likely induce additional bias in estimates of gosling growth rates.

We used *f* statistics (*f*), or the portion of the posterior distribution on the same side of zero as the mean, to interpret the probability of slopes being positive or negative. This statistic is reported with all model results.

### 2.6 Grazing lawn analysis

To examine long‐term trends in the abundance of *C. subspathacea* at our study site, we constructed mixed‐effects models in JAGS. We modeled the extent of *C. subspathacea* during each year (*P*_C.sub,_ *_t_*) as random variation around a yearly mean (*µ_t_*_,extent_) with variance ($\sigma_{C.\text{sub}}^{2}$).

$$P_{C.\text{sub},t}∼\text{normal(}\mu_{t,\text{extent}},\sigma_{C.\text{sub}}^{2}),$$

$$\mu_{t,\text{extent}}=\alpha_{t,\text{extent}}+\beta_{\text{extent}}*\text{year}.$$

We assumed that each photo we examined comprised a random sample of the total spatial extent of *C. subspathacea* at the colony, where we modeled the number of points placed on grazing lawn within each photo as a binomial trial, in which *y_i,t_* was the number of points composed of grazing lawn in the *i*th photo in the *t*th year, *P*_c.sub,_ *_t_* was the probability a point was grazing lawn in the *tth* year, and *K_i_* was the number of sampled points in the *i*th photo in the *t*th year.

$$y_{i,t}∼\text{binomial(}K_{i,t},P_{C.\text{sub},t}).$$

### 2.7 Behavior analysis

To examine behavior during brood rearing, we constructed mixed‐effects binomial models in JAGS, where the response was the number of times a behavior was exhibited by adult males, adult females, or goslings (group) during an observation bout (*n*) given the probability of that behavior (θ), and the number of trials was the number of observations (*N*) during each observation bout,

$$n_{{}_{i,\text{group}}}∼\text{binomial}\left(N_{i},\theta_{t,\text{group}}\right).$$

We modeled each behavior separately for adult males and females, and goslings as a random effect around a group‐specific mean $(\mu_{\theta ,t,\text{group}}),$

$$\theta_{t,\text{group}}∼\text{normal}\left(\mu_{\theta ,t,\text{group}},\sigma_{\theta ,\text{group}}^{2}\right),$$

where we modeled the mean as a linear trend across years

$$\mu_{\theta ,t,\text{group}}=\alpha_{t,\text{group}}+\beta_{t,\text{group}}*\text{year}.$$

We acknowledge that behaviors were not independent of one another, because the probabilities of these behaviors must sum to 1, potentially having minor impacts on the estimates of variances of each behavior.

### 2.8 Correlative analysis

We estimated the correlations among the predicted parameter values from the previously developed models by sampling the posterior distributions from each model and estimating the correlations between the posterior distributions using linear regressions. Posterior outputs for response variables from grazing lawn, gosling mass, gosling walking, and gosling resting models were sampled 300 times for each year where data were collected for both variables (1991–2014) to maintain variation in parameter values as predicted by the previously models. Gosling resting and walking behaviors were chosen to be modeled as they had shown considerable temporal changes and are likely related to observed trends in gosling mass.

## 3 RESULTS

**Figure 1:**
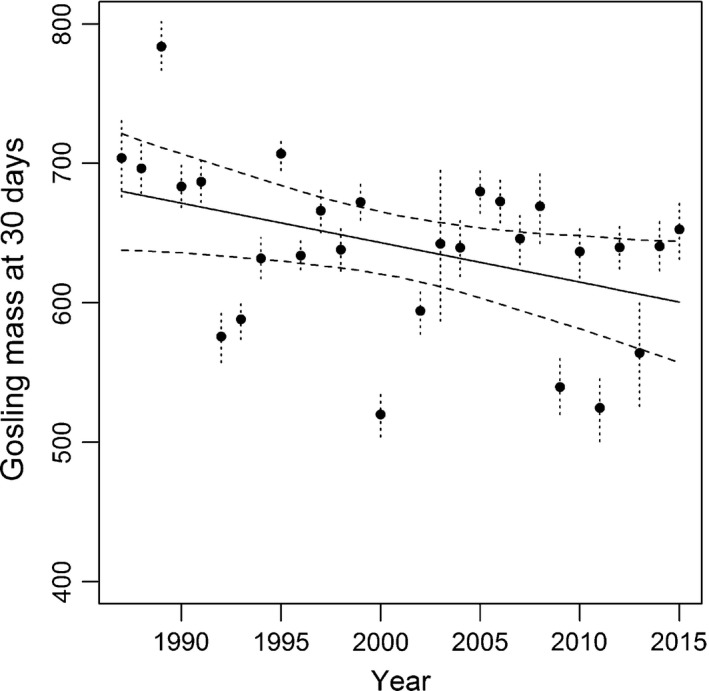
Annual estimates and 95% credible intervals (dashed) of model‐predicted mass at 30 days black brant goslings captured on brood‐rearing areas near the Tutakoke River colony, 1987–2015. Linear trend (solid) and 95% credible intervals for linear trend (dashed) are also shown. No estimates were available in 2001

**Figure 2:**
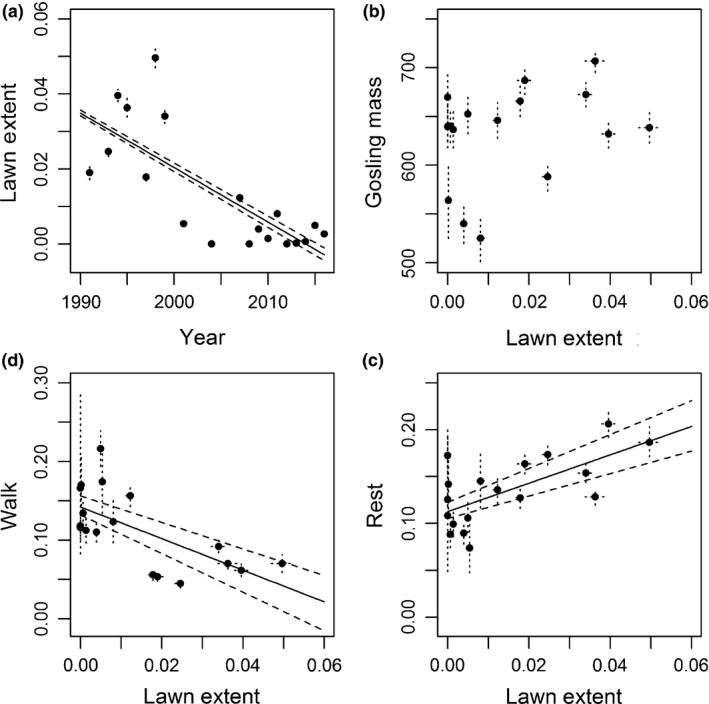
(a) Annual estimates and 95% credible intervals (dashed) of model‐predicted proportion of the colony covered by *Carex subspathacea*, the primary forage of juvenile brant, 1991–2016. Effects of annual proportion of *C. subspathacea* extent on annual estimates of black brant gosling (b) the predicted mass at 30 days, (c) the proportion of time resting, and (d) the proportion of time walking. Dotted lines on graphs indicated 95% credible intervals for estimates of both x‐ and y‐axis variables. No estimates were available for 1992, 1996, 2000, 2002, 2003, 2005, or 2006

**Figure 3:**
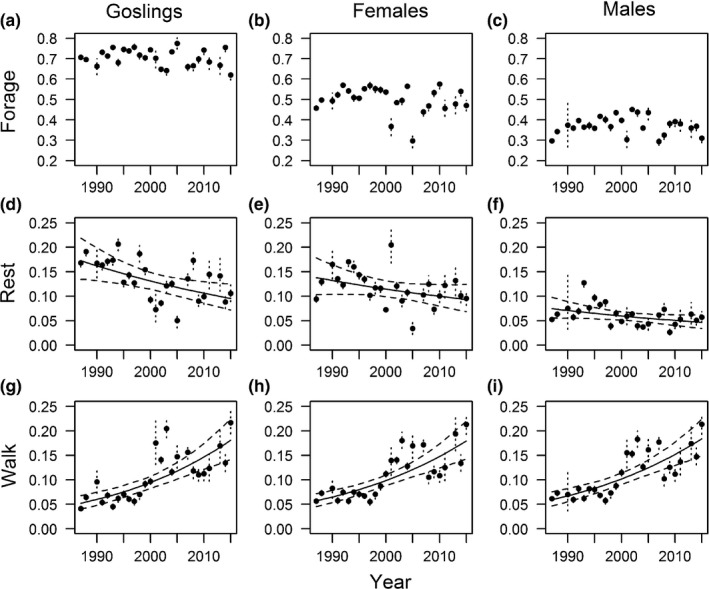
Annual estimates and 95% credible intervals (dashed lines) of model‐predicted proportion of time spent (a, b, c) foraging, (d, e, f) resting, and (g, h, i) walking for (a, d, g) juvenile, and adult (b, e, f) female, and (c, h, i) male brant on the Tutakoke River colony, 1987–2015. Linear trends (solid) and 95% credible intervals for linear trend (dashed) are also shown. No estimates were available in 1989, 2006, or 2012

**Figure 4:**
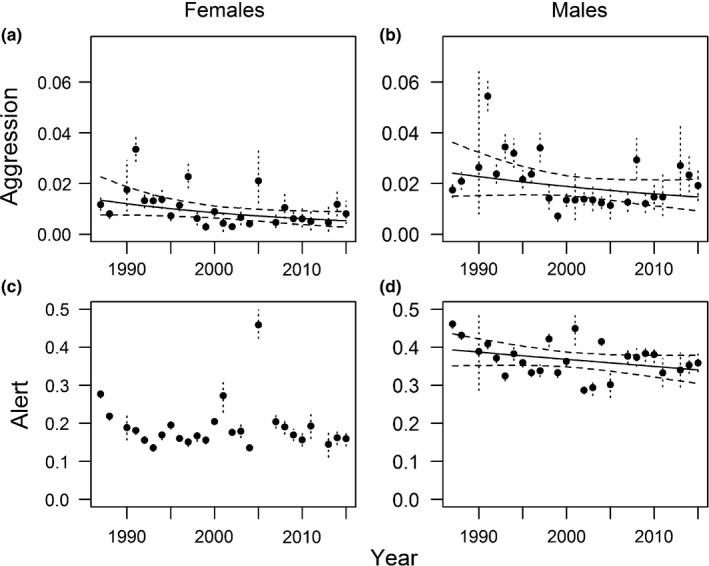
Annual estimates and 95% credible intervals (dashed lines) of model‐predicted proportion of time spent on aggression and alert behaviors for adult female and male brant at the Tutakoke River colony, 1987–2015. Linear trends (solid) and 95% credible intervals for linear trend (dashed) are also shown. No estimates were available in 1989, 2006, or 2012

Gosling mass at 30 days was highly variable among years, but generally declined between 1987 and 2015 (*β* = −0.096; 95% CRI = −0.191, −0.016; *f* = 1.000; Figure 1, 2, 3, 4; Table 1). The proportion of the landscape covered by *C. subspathacea* also declined substantially from 1991 through 2016 (*β* = −0.194; 95% CRI = −0.350 to −0.037; *f* = 0.992; Figure 1, 2, 3, 4a; Table 2).

**Table 1:** Parameter estimates of linear trends from models estimating the daily growth rate (g/day) for black brant goslings captured on brood‐rearing areas near the Tutakoke River Brant colony, Alaska, USA, 1987–2014

Estimate	Parameter	
	*α*	*β*
*μ*	21.422	−0.100
*σ*	0.0797	0.045
2.5% CRI	19.964	−0.191
97.5% CRI	22.991	−0.016
*f*	1.000	0.988

Note

Mean growth rate (*μ*), standard deviation (*σ*), 2.5% and 97.5% credible intervals (CRI), and the proportion of the posterior distribution on the same side of zero as the mean (*f*) are given

**Table 2:** Parameter estimates of linear trends from models estimating the aerial extent of grazing lawn near the Tutakoke River Brant colony, Alaska, USA, 1991–2016

Parameter estimates	Parameter	
	*α*	*β*
*μ*	−3.126	−0.191
*σ*	1.339	0.079
2.5% CRI	−5.741	−0.355
97.5% CRI	−0.443	−0.032
*f*	0.988	0.994

Note

Mean aerial grazing lawn extent (*μ*), standard deviation (*σ*), 2.5% and 97.5% credible intervals (CRI), and the proportion of the posterior distribution on the same side of zero as the mean (*f*) are given

We detected no substantial trends from 1987 to 2015 in mean proportion of time devoted to foraging for goslings (*β* = −0.005; 95% CRI = −0.015, 0.005; *f* = 0.831; *μ* = 0.705, Figure 1, 2, 3, 4; Table 3), females (*β* = −0.005; 95% CRI = −0.019, 0.008; *f* = 0.795; *μ* = 0.501; Figure 1, 2, 3, 4; Table 3), or males (*β* = 0.000; 95% CRI = −0.011, 0.010; *f* = 0.513; *μ* = 0.372; Figure 1, 2, 3, 4; Table 3). In contrast, time devoted to locomotion increased for goslings (*β* = 0.050; 95% CRI = 0.032, 0.068; *μ* = 0.109; *f* = 1.000; Figure 1, 2, 3, 4; Table 3), females (*β* = 0.046; 95% CRI = 0.029, 0.060; *f* = 1.000, *μ* = 0.111; Figure 1, 2, 3, 4; Table 3), and males (*β* = 0.045; 95% CRI = 0.030, 0.060; *f* = 1.000; *μ* = 0.062; Figure 1, 2, 3, 4; Table 3). Resting behaviors decreased for goslings (*β* = −0.024; 95% CRI = −0.041, −0.007; *f* = 0.994; *μ* = 0.135; Figure 1, 2, 3, 4; Table 3), adult females (*β* = −0.016; 95% CRI = −0.036, 0.003; *f* = 0.942; *μ* = 0.118; Figure 1, 2, 3, 4; Table 3), and adult males (*β* = −0.018; 95% CRI = −0.038, 0.001; *f* = 0.961; *μ* = 0.114; Figure 1, 2, 3, 4; Table 3).

**Table 3:** Beta (*β*) parameter estimates of linear trends from models estimating the proportion of time spent foraging, resting, and walking for juvenile, female, and male brant on the Tutakoke River Brant colony, Alaska, USA, 1987–2015

	*β* estimate	Behavi		
		Fage	Walk	Rest
Goslings	*μ*	−0.005	0.050	−0.024
	*σ*	0.005	0.009	0.009
	2.5% CRI	−0.015	0.032	−0.041
	97.5% CRI	0.005	0.068	−0.007
	*f*	0.831	1.000	0.994
Females	*μ*	−0.005	0.046	−0.016
	*σ*	0.007	0.008	0.010
	2.5% CRI	−0.019	0.029	−0.036
	97.5% CRI	0.008	0.060	0.003
	*f*	0.793	1.000	0.942
Males	*μ*	0.000	0.045	−0.018
	*σ*	0.006	0.008	0.010
	2.5% CRI	−0.011	0.030	−0.038
	97.5% CRI	0.010	0.060	0.001
	*f*	0.513	1.000	0.961

Note

Mean proportion of time spent on a given behavior (*μ*), standard deviation (*σ*), 2.5% and 97.5% credible intervals (CRI), and the proportion of the posterior distribution on the same side of zero as the mean (*f*) are given

Aggressive behaviors declined for both adult females (*β* = −0.34; 95% CRI = −0.071, 0.001; *f* = 0.97; *μ* = 0.010; Figure 1, 2, 3, 4; Table 4) and adult males (*β* = −0.018; 95% CRI = −0.044, 0.008; *f* = 0.915; *μ* = 0.021; Figure 1, 2, 3, 4; Table 4). Alert behaviors declined for males (*β* = −0.008; 95% CRI = −0.019, 0.003; *f* = 0.92; *μ* = 0.368; Figure 1, 2, 3, 4; Table 4), but did not change for females (*β* = −0.005; 95% CRI = −0.023, 0.013; *f* = 0.686; *μ* = 0. 191; Figure 1, 2, 3, 4; Table 4). Goslings spent little time in aggressive (*μ* ~ 0.000) or alert (*μ* = 0.006) behaviors, and so, trends were not included in further analyses.

**Table 4:** Beta (*β*) parameter estimates of linear trends from models estimating the proportion of time spent on aggression and alertness for adult female and male brant at the Tutakoke River Brant colony, Alaska, USA, 1987–2015

	*β* estimate	Behavi	
		Aggression	Alert
Females	*μ*	−0.034	−0.005
	*σ*	0.018	0.010
	2.5% CRI	−0.071	−0.023
	97.5% CRI	0.001	0.013
	*f*	0.97	0.686
Males	*μ*	−0.018	−0.008
	*σ*	0.013	0.005
	2.5% CRI	−0.044	−0.019
	97.5% CRI	0.008	0.003
	*f*	0.915	0.92

Note

Mean proportion of time spent on a given behavior (*μ*), standard deviation (*σ*), 2.5% and 97.5% credible intervals (CRI), and the proportion of the posterior distribution on the same side of zero as the mean (*f*) are given

### 3.1 Correlations between grazing lawn, behaviors, and gosling mass

Our results indicated a 76.97% chance of a positive trend between gosling mass and grazing lawn (*β* = 939.58, *f* = 1.000, Figure 1, 2, 3, 4b) from 1991 to 2014. However, we also saw dramatic variation in colony size during this time and were unwilling to fit models with multiple covariates to estimate colony densities and their correlations with gosling mass given only 16 years of shared data. Positive correlations with gosling resting (*β* = 1.49, *f* = 1.000, Figure 1, 2, 3, 4c) and negative correlations with walking (*β* = −2.04, *f* = 1.000, Figure 1, 2, 3, 4d) behaviors were observed.

## 4 DISCUSSION

### 4.1 Demographic implications of long‐term changes in vegetation and growth rates for brant

We observed long‐term declines in gosling growth rates, indicating declining food abundance in gosling diets (Hupp et al., 2017; Sedinger et al., 2016). Correlations between gosling mass at 30 days and grazing lawn extent, as well as the evidence of declines in the mass of adult female brant at the beginning of their wing feather molt (16 days after their clutches hatch) since 1992 (Van Dellen, 2016) support the hypothesis that declining forage abundance has considerably influenced gosling mass. While results of correlations between gosling mass declines and grazing lawn declines indicated only a 76.97% chance of a positive relationship between grazing lawn extent and gosling growth, the period studied had substantial variation in the size of breeding colonies densities (Van Dellen, 2016; Person et al., 2003) that may have influenced availability of food for individuals. Declines in grazing lawn extent began occurring in the late 1990s and early 2000s, concurrent with periods of increased predation on brant nests by arctic foxes and molt migration away from the breeding colony and associated brood‐rearing areas (Van Dellen, 2016; Person et al., 2003; Uher‐Koch et al., unpublished data) resulting in lower brood densities. Thus, grazing lawn declines appear to stem from, at least in part, reduced grazing caused by declines in the nesting population. Concurrently, declines in colony size potentially reduce competition for food within a given breeding season and allowing for increased gosling growth rates at a given grazing lawn extent.

As these reductions in forage abundance occur, decreases in gosling growth rates and associated declines in first‐year survival exacerbate short‐term effects of fox predation, causing recruitment rates to move below levels required to replace adult mortality since the late 1990s (Sedinger et al., 2016). As numbers of nests have decreased (Wilson, 2016), relatively lower rates of predation have greater effects on the recruitment process because, (a) a given number of destroyed nests represent a greater proportion of nests, and consequently recruits, and (b) declining aerial extent of grazing lawn reduces per capita food availability even in the face of declining population size. The result is a trophic cascade, where negative effects from a primary predator are disrupting the positive feedback loop that exists between an herbivore and its food plant.

During an increase in colony size during the late 1980s and early 1990s, an increase was observed in time spent foraging by adult brant in both our analyses as well as previous research (Sedinger, Eichholz, et al., 1995), which was attributed to reduced standing crop of vegetation, associated with increased brood density. Since then, however, changes in adult and gosling behavior have been unable to mitigate the effects of declines in resource availability as grazing lawn extent has dropped below levels necessary to support a positive nutrient balance (Herzog & Sedinger, 2004). These patterns are critically important, as this grazing maintains the spatial extent of *C. subspathacea*, which when not grazed, reverts to lawns from a less nutritious and longer growth form Person et al. (2003). As a consequence, individuals from cohorts that experienced reduced availability of high‐quality grazing lawn may have lower juvenile survival and future probability of breeding (Sedinger, Flint, et al., 1995).

Though not directly addressed in analyses here, increasing evidence of phenological mismatches in arctic and subarctic systems suggest possible disparities between peak nutrient availability and hatch date that may also have contributed to decreased gosling mass (Ross et al., 2017) and recruitment (Clausen & Clausen, 2013). If present, this phenomenon would indicate that decreased nutrient acquisition by goslings is a larger consequence of a multifaceted issue comprised of decreased nutrient availability in both time and space. The complexity of the problem could factor into the inability of brant to mitigate changing environmental conditions, even with adjustments in foraging behavior, resulting in long‐term declines in gosling growth.

### 4.2 Contributions to long‐term shifts in gosling behavior

Foraging behavior for goslings remained relatively constant over the past 29 years, while their locomotion increased and resting behavior declined. Growth rates of goslings declined over the same period and were substantially below the maximum possible when food is not limiting (Hupp et al., 2017; Sedinger et al., 2001). Because size at fledging is directly predictive of first‐year survival (Cooch, Jefferies, Rockwell, & Cooke, 1992; Owen & Black, 1989; Sedinger & Chelgren, 2007) and adult fitness (Riecke, Leach, Gibson, & Sedinger, 2018; Sedinger, Flint, et al., 1995; Sedinger et al., 2004), declining growth rates suggest that availability of sufficient quality food limits growth rates as individuals try to maximize growth by foraging as much as possible. Thus, it remains to be explained why brant broods did not increase foraging time in response to poorer growth conditions over the duration of this study.

Our primary hypothesis concerns earlier work with captive brant goslings that found that foraging time declined substantially at very low biomass of *C. subspathacea* (Herzog & Sedinger, 2004). While we observed an increase in foraging behavior during the late 1980s and early 1990s when per capita foraging abundance was also relatively low, aerial extent of *C. subspathacea* grazing lawns has dramatically declined since that period. We propose that biomass of grazing lawns has sufficiently decreased that brant goslings could no longer efficiently forage on remaining grazing lawns, where locating and traveling to grazing lawns has become a more time‐consuming process and reduces time available to forage. Increased locomotory behavior is, thus, a response to reduced foraging efficiency and decrease in the spatial extent of grazing lawn, where patches of grazing lawn with sufficient biomass to support grazing by goslings may be more difficult to find. The decline in the proportion of time spent resting corresponds with increases in locomotion, where increases in time allocated to searching behavior decrease the amount that can be spent on other behaviors, such as resting. Additionally, reductions in time spent resting may be a result of reduced digestive bottlenecks (Kersten & Visser, 1996; Sedinger & Raveling, 1988). With correlations between lawn extent and resting behaviors, we hypothesize that more time spent locating and walking to grazing lawns allows for longer periods of digestion, thus less time needed to rest.

This decrease in forage efficiency may also tie changes in gosling mass to behavioral trends more directly. With increased time engaged in locomotory behaviors and decreased time resting, goslings may have increased energetic demands that necessitate accumulated nutrients to be taken away from growth processes (Case, 1978). Additionally, forage consumed by brant adults and goslings may not exclusively be the shorter and more nutritious growth form of *C. subspathacea*. Brant have been observed foraging on the longer growth form of the sedge when high densities of broods on the YKD reduced availability of short meadows (Person et al., 2003). While declines in forage availability examined here result from reduced grazing pressure, similar shifts in dietary specialization may be occurring. If so, goslings are ingesting less nutritious food and further limiting time spent foraging on high‐quality grazing lawns. These issues compound the problem of decreasing nutrient availability and may factor into the inability of brant to alleviate issues created by grazing lawn declines.

### 4.3 Variation in adult behavior

Adult geese face nutritional demands following hatch and must restore depleted lipid and protein invested in reproduction before the fall migration (Ankney, 1984; Ankney & MacInnes, 1978; Sedinger & Alisauskas, 2014). The importance of restoring depleted nutrients by adults creates potential trade‐offs between brood care and foraging (Sedinger & Raveling, 1990) during brood rearing because adults simultaneously undergo molt, which may cause nutritional stress and influence care for precocial offspring (Owen & Ogilvie, 1979; McLandress & Raveling, 1981; Van Dellen, unpublished data). We observed declines in aggression for both females and males, concurrent with long‐term declines in colony size (Wilson, 2016) and nest survival (Van Dellen, 2016) that have resulted in reduced brood densities at Tutakoke River colony (Sedinger et al., 2016). Families of geese are known to compete for and defend specific foraging patches (Black, Carbone, Wells, & Owen, 1992; Mulder et al., 1995). If a brood maintains or gains access to a particular grazing lawn, both adults and goslings may decrease energy spent locating and traveling to different grazing lawns and increase forage efficiency (Mulder et al., 1995). So, decreases in aggression may be related to the declines in densities of broods, although reductions in the aerial extent of grazing lawns may have partially maintained the level of aggressive interactions by concentrating broods on smaller remaining areas of grazing lawn.

Adult males also spent less time in alert behavior in later years of the study (Figure 1, 2, 3, 4). These declines may represent declining investment in parental care by adult males (Lazarus & Inglis, 1978; Schindler & Lamprecht, 1987), where males trade‐off time spent on alert and foraging behaviors, and could contribute to lower prefledging survival of offspring (Nicolai & Sedinger, 2012). Adult locomotive behaviors increased substantially while resting behaviors declined, mirroring shifts in gosling behavior. These trends further support our hypothesis that pervasive habitat change has influenced both gosling growth and adult behavioral patterns.

We also observed sex‐specific differences, consistent with previous research showing higher rates of aggressive and alert behaviors by males (Gauthier & Tardif, 1991; Sedinger & Raveling, 1990), associated with increased nutritional requirements of females to regain mass lost during egg laying and incubation (Gauthier & Tardif, 1991; Lazarus & Inglis, 1978; Sedinger & Raveling, 1990).

## 5 CONCLUSION

We show that both behavior and growth rates of brant broods have responded to, but were unable to mitigate, substantial changes in food abundance over a 30‐year period. Declines in gosling growth may have contributed to lower first‐year survival (Sedinger & Chelgren, 2007) and subsequent recruitment (Sedinger et al., 2016). In contrast to our original prediction, we did not identify an increase in foraging time, but identified increases in locomotory behavior. We primarily attribute this to sedge biomass in grazing lawns being too low to support efficient foraging by brant (Herzog & Sedinger, 2004). Changes in sedge biomass and extent have resulted in part from reduced grazing by brant (Sedinger et al., 2016), which in turn is strongly affected by nest predation by arctic foxes. Thus, the fox‐brant‐grazing lawn system appears to be experiencing a trophic cascade that threatens the local brant population and that brant are unable to compensate for by modifying their behavior. This inability to mitigate changes in food abundance has resulted in declines of breeding brant at all major colonies on the Yukon–Kuskokwim Delta (Wilson, 2016).

## CONFLICT OF INTEREST

None declared

## AUTHOR CONTRIBUTIONS

JSS conceived the long‐term avian monitoring program and has led data collection efforts through the course of the project. BTP and BDU‐K led habitat classification of aerial photographs and videography. MGL and TVR conceived and led the analysis, with important contributions from CRA and JSS. MGL led the writing of the manuscript, with important contribution from all coauthors.

## Data Availability

Data are available on the Dryad Digital Repository (https:https://doi.org/10.5061/dryad.vb653hv). Data used in this study are publicly available via the U.S. Geological Survey Alaska Science Center (Uher‐Koch, 2019).
